# Relationship between plasma level of vitamin D and post operative atrial fibrillation in patients undergoing CABG

**DOI:** 10.12669/pjms.324.10587

**Published:** 2016

**Authors:** Kamran Shadvar, Fariba Ramezani, Sarvin Sanaie, Taher Entezari Maleki, Babak Kazemi Arbat, Bahman Nagipour

**Affiliations:** 1 Kamran Shadvar, Assistant Professor, Fellowship of Critical Care Medicine, Department of Anesthesiology, Faculty of Medicine, Tabriz University of Medical Sciences, Tabriz, Iran; 2 Fariba Ramezani, General Physician, Faculty of Medicine, Tabriz University of Medical Sciences, Tabriz, Iran; 3 Sarvin Sanaie, Assistant Professor of Nutrition, Tuberculosis and Lung Disease Research Center, Tabriz University of Medical Sciences, Tabriz, Iran; 4 Taher Entezari Maleki, Assistant Professor, Department of Clinical Pharmacy, Faculty of Pharmacy, Tabriz University of Medical Sciences, Tabriz, Iran; 5 Babak Kazemi Arbat, Associate Professor, Fellowship of Electrophysiologic Study, Department of Cardiology, Faculty of Medicine, Tabriz University of Medical Sciences, Tabriz, Iran; 6 Bahman Nagipour, Assistant Professor, Fellowship of Cardiac Anesthesia, Tabriz University of Medical Sciences, Tabriz, Iran

**Keywords:** Atrial fibrillation, CABG, Vitamin D

## Abstract

**Objective:**

Atrial fibrillation is the most common arrhythmia after cardiac surgery. Several studies have shown the impact of vitamin D on heart disease; however, there have been few studies for the incidence of AF and its relationship with vitamin D levels. According to the different results of these studies, we decided to evaluate the relation of plasma levels of vitamin D and postoperative atrial fibrillation in patients undergoing coronary artery bypass surgery (CABG).

**Methods:**

This cross-sectional study was performed on 50 patients after CABG surgery. Simple random sampling was done. Twenty five patients who developed AF within 48 hours after CABG with Cardiopulmonary bypass (CPB) were enrolled in the case group and 25 patients who did not develop AF within 48 hours after CABG with CPB were enrolled in the control group. Plasma levels of vitamin D in both groups of patients were recorded. Collected data were analyzed by the SPSS software version 17.

**Results:**

There was no significant difference in terms of demographic characteristics, comorbidities, lipid profile and kidney function between two groups. The mean plasma level of vitamin D was 27.4 ± 2.22 ng/ml in the case group and was 28.2 ± 1.18 ng/ml in the control group it (p= 0.803).

**Conclusions:**

Plasma levels of vitamin D were almost the same in both groups and there was no statistically significant difference between the groups with and without atrial fibrillation following CABG.

## INTRODUCTION

Atrial fibrillation (AF) is the most common sustained arrhythmia and has a major impact on the morbidity and mortality of patients.^1^ The risk of AF in general population after the age of 40 is 26%. It is estimated that approximately 2.3 million adults in the United States have AF and this number will increase to 6.5 to 15.9 million by 2050.^2^,^3^ AF is an important risk factor for stroke and systemic embolism.^4^ This disorder increases mortality rate caused by stroke to five times and doubles the mortality in patients with cardiac disease compared with the control group.^5^,^6^

The risk of stroke in patients with AF increases with age.^7^ Prevention of AF-related stroke can be done by modifying or changing the overall cardiovascular risk factors and the use of systemic anticoagulation drug therapy.^8^ Atrial fibrillation is the most common arrhythmia after heart surgery. This condition is an important factor for prolonged hospitalization and incidence of neurological and kidney complications. The prevalence of atrial fibrillation after cardiac surgery varies from 30% to 50% and in patients undergoing non-cardiac surgeries, it has been reported to be 12-74%.^1^,^2^ Several factors affect the incidence of atrial fibrillation after surgery and inflammatory and oxidative factors are some of the most important ones. Some studies indicate oxidative damage in the heart tissue of patients with AF.^3^,^9^

Other studies show an increase in serum myocardial oxidative markers such as Peroxynitrite and Superoxide in atrial fibrillation after surgery.^10^,^11^ Antioxidants such as vitamin C, N- acetylcysteine and statins lower serum levels of oxidants.^4^ In recent years, there has been a growing interest toward the role of vitamin D on cardiovascular health.^12^,^13^ Lack of vitamin D is related to hypertension,^14^,^15^ stroke,^16^,^17^ Myocardial Infarction^16^ and other cardiovascular related diseases such as diabetes mellitus.^18^ The main sources of vitamin D are sunlight, diet and supplements. Plasma 25-hydroxyvitamin D level is the most accurate method to assess vitamin D deficiency.^7^ A plasma 25-hydroxy vitamin D level between 32 to 50 ng/ml is considered as the normal range.^19^

Studies have shown that vitamin D, due to its antioxidant properties is effective in reducing the incidence of postoperative AF. Vitamin D regulates the renin-angiotensin-aldosterone system is negative fashion and has antioxidant effects that reduce oxygen free radicals in the atria, which are associated with inflammation and the production of proarrhythmic materials^20^.

Due to limitations of previous studies we decided to investigate the relationship between plasma levels of vitamin D and postoperative atrial fibrillation in patients undergoing coronary artery bypass surgery.

## METHODS

After approval of the Ethics Committee of Tabriz University of Medical Sciences and obtaining informed consent, patients scheduled for CABG surgery under CPB and hospitalized during 2015 in the Shahid Madani Hospital of Tabriz, were enrolled in this cross sectional study. Simple random sampling was done. The sample size was calculated using Demir et al.^21^ study with 95% confidence level and statistical power of 80% and alpha of 0.05 with 21 patients in each group which was increased to 25 patients in each group.

Inclusion criteria were patients undergoing CABG under CPB, age 40 to 60 years and willing to participate in the study. Exclusion criteria were vitamin D supplementation in recent month, patients with renal failure, parathyroid disorders, pregnant women, disability, sarcoidosis, rickets and hypophosphatemia, heart valve surgery, Rheumatic and Nonrheumatic Valvular Heart Disease (≥moderate), Redo operation and patients with a history of AF.

Twenty five patients who underwent CABG under CPB and developed AF within 48 hours after surgery were enrolled in the case group and 25 patients who underwent CABG under CPB and did not develop AF within 48 hours after surgery were enrolled in the control group. Plasma levels of vitamin D, calcium and magnesium were measured in both groups; then average vitamin D levels in two groups were compared and the relationship between vitamin D and AF was evaluated. To evaluate the incidence of AF, all patients in both groups underwent Holter monitoring for 48 hours postoperatively and holter results were interpreted by a cardiologist.

All demographic characteristics, underlying medical conditions, medications, history of surgery, duration of surgery, CPB and Aortic cross-clamp time were noted by the investigators as a checklist for each patient.

The data were analyzed by descriptive statistics (frequency - percent), independent t test or Mann-Whitney and if need regression model using SPSS TM software version 17. The p value less than 0.05 is considered statistically significant.

## RESULTS

In this study, 25 patients were enrolled in each group. There was no significant difference in terms of demographic characteristics including age, sex, BMI and education between two groups (Table-I). There were also no significant differences between the two groups in terms of history of an underlying medical condition such as diabetes, hypertension, myocardial infarction, hyperlipidemia, and smoking and alcohol (Table-II). The mean plasma level of vitamin D was 27.4±2.22IN AF group and 28.2±1.18 in control group, which did not have any significant difference between the two groups. (p=0.803) (Table-II).

**Table-I:** Demographic variables in both groups.

	AF Group	Control group	P value
Age (year)	3/0±69	8/0±72	0.27
Male	13	13	
Female	12	12	
Low Education (%)	5/0±25	3/0±33	0.08
Smoke (%)	1/0±14	2/0±17	0.12
BMI	4/0±26	2/0±27	0.17

**Table-II:** The results of the study variables in both groups.

	AF Group	Control group	P-value
Operation time (hours)	0.75±5.26	0.71±5.53	0.474
CPB time(min)	31.64±85.27	38.03±95.93	0.282
DM	14	15	0.73
HTN	19	16	
MI	5	5	
hyperlipidemia	4	4	
alcohol abuse	3	4	
LDL (mg/dl)	6/5±05/106	7/5±05/108	761/0
HDL (mg/dl)	2/3±32/49	3/3±22/50	923/0
TG (mg/dl)	6/7±1/195	4/6±1/185	031/0
Cholesterol(mg/dl)	8/7±3/198	5/6±2/188	032/0
25(OH)vitD(ng/ml) (male)	22/2±4/27	18/1±2/28	0.803
25(OH)vitD(ng/ml) (female)	26/3±2/17	27/5±1/06	
SBP (mmHg)	142	145	88/0
DBP(mmHg)	75	75	1
Hypercalcemia %	11	44	08/0
Hypocalcemia %	2	18	09/0
Urea (mg/dl)	19.85±5.99	16.90±5.16	0.110
Cr (mg/dl)	1.22±0.23	1.07±0.19	0.017
Na (mEq/l)	143.2±3.48	142.5±2.64	0.438
K (mEq/l)	4.34±0.31	4.46±0.27	0.195
Mg (mg/dl)	1.68±0.32	1.70±0.29	0.834
T3 ng/dl	112	119	0.11
T4ug/dl	9.3	10.3	0.12
TSH U/ml	1.2	1.8	0.09

There was no statistically significant difference between two groups in terms of duration of surgery and CPB time. In the case of cholesterol and triglycerides, a significant relationship was observed between two groups (p=0.031, p=0.032); While no statistically significant relationship was observed in terms of LDL and HDL between the groups. (p=0.923, p=0.761)

Creatinine levels was 1.22±0.23 mg/dl in AF group and 1.07±0.19 mg/dl in the control group which a statistically significant difference was observed between the groups. (p=0.017) (Table-II). Blood urea level in AF group was 19.85±5.99 mg/dl and in the control group was 16.90±5.16 mg/dl that there was no statistically significant difference between the groups. (p=0.110). In general, except for blood creatinine, the rest of parameters, such as urea, sodium, potassium and magnesium had no statistically significant difference between two groups.

**Fig.1:**
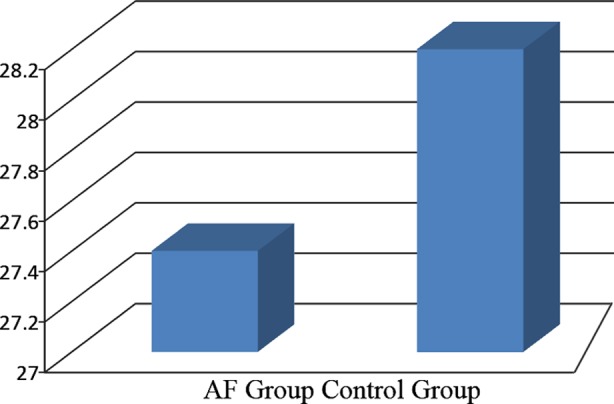
Average levels of vitamin D in patients with and without atrial fibrillation after CABG.

## DISCUSSION

Nowadays, lack of vitamin D can be seen everywhere and many people are suffering from this deficiency. A study showed that blood levels of 25-hydroxyvitamin D less than 30 ng/ml is associated with an increased risk of heart attack. In this study, heart attack risk in men with plasma level of 25 hydroxy-vitamin D>30 ng/ml reduced by 50% regardless of other cardiovascular risk factors.^1^,^2^ Death from cardiovascular diseases has been linked to vitamin D deficiency. American Heart Association has published the results of an investigation that shows the relationship between vitamin D deficiency and peripheral vascular diseases; reduction of each ng/ml of vitamin D, there will be 35% higher risk of suffering from disease.^3^ The results of our study showed no significant differences between the groups with and without atrial fibrillation undergoing CABG. In this regard few studies have been done that the results of present study are generally consistent with the results of previous research in this area.

A study by Qayum et al. on 258 patients with AF showed no relationship between vitamin D levels and type of AF. In addition, no link was found between vitamin D deficiency and ischemic heart disease, heart attack or acute myocardial infarction. Lack of vitamin D, was significantly associated with age and gender. They concluded that although the beneficial effect of vitamin D on cardiovascular disease has been discovered, no association between vitamin D deficiency and risk for AF or its type was found. Therefore, further investigation is needed to determine whether vitamin D supplementation improves cardiovascular outcomes in patients with AF.^22^

In another study conducted by Korantzopulos, effect of administration of oral vitamin D in reducing the recurrence of atrial fibrillation was evaluated in 44 patients who got continuous AF under electric cardioversion. Duration of developing continuous AF was evaluated by ECC investigation. Patients were divided in two groups of case and control. The case group received two grams of oral vitamin D 12 hours before cardioversion and then 500 mg twice daily for seven days. The levels of WBC, CRP were examined in the first, the third and the seventh days after treatment. A week after cardioversion incidence of atrial fibrillation in intervention and control groups was respectively 5/4% and 3/36%.(p=0.024), so they found evidence of the effectiveness of vitamin D in reducing the recurrence of atrial fibrillation^23^ The results of this study were not consistent with the results of our study which can be due to the baseline plasma level of vit D. In the above mentioned study, patients were already taking vitamin D but in our study, the average of vitamin D level was generally low and the patients had some degrees of vitamin D deficiency.

Reinstra et al. in a 9-year follow-up of 425 participants (15%) with AF concluded that vitamin D status is not related with AF and their data showed that vitamin D deficiency does not increase the incidence of AF.^24^ Results of this study were consistent with our results.

Chen et al.^25^ enrolled 162 patients with chronic AF and 160 healthy individuals without AF in a study and measured their serum 25-hydroxyvitamin D levels. They showed that plasma 25 (OH) D level was significantly lower in AF group compared to non-AF group. Hs-CRP level was significantly higher in AF group compared to non-AF. The mean diameter of the left atrium in AF group was significantly larger than non AF group. Plasma level of 25(OH) D showed an inverse relation with the diameter of the left atrium, pulmonary artery systolic pressure and levels of hs-CRP. Logistic regression analysis determined that low level of 25(OH) D is significantly associated with AF. Patients with vitamin D deficiency suffering from AF were two times higher than those with normal levels of vitamin D. They concluded that low vitamin D levels were associated with AF and might even cause it.^25^ Results of this study do not match with our study because the results of our study showed no significant difference between the groups with and without atrial fibrillation under CABG in terms of vitamin D levels the cause of which can be considered as the differences in the nature and type of the selected patients.

Demir et al. enrolled 102 patients with chronic non-valvular AF with no other cardiovascular disease (group 1) and 96 patients with AF associated with mitral valve disease (group 2) and 100 age matched patients with sinus rhythm as the control group. Group 1 patients had lower vitamin D levels than those in group 2 and the control group. In groups 1 and 2, the diameter of the left atrium and pulmonary artery systolic pressure was higher than the control group. They concluded that there is a significant relationship between the lack of vitamin D and non-valvular AF.^25^ The results of our study are not consistent with the study of Demir.^21^ It can be due to the larger sample size of this study or confounding variables. According to the results of our study regarding the parameters of T3, T4 and TSH, there was no significant association between the two groups. In studies of Pentagon and Housenderg there was no significant difference between the parameters of the thyroid and vitamin D levels. The results of the both studies, similar to our studies, emphasized the issue.

Moreover, no significant difference was observed between the duration of the study and underlying medical condition in the two groups. According to the results, the average vitamin D levels were reported low in both groups but no statistically significant difference was observed between the groups with and without atrial fibrillation in this study. It was performed only in one ICU and only in CABG patients, hence we cannot generalize the results of this study for all patients.

### Limitation of the study

We did not measure cardiac function of patients after surgery with echocardiography and we did not collect patients EF (ejection fraction) before surgery. Another limitation is that our study has Low sample size. In this study we only evaluated the relationship between the plasma level of Vit D and postoperative CABG and we did not evaluate the relationship based on the insufficiency or deficiency of Vit D level.

## Conclusion

According to the results of this study it can be concluded that the plasma levels of vitamin D was similar in both groups and no statistically significant differences were observed between the groups with and without atrial fibrillation following coronary artery bypass surgery. Due to the fact that a clear difference between plasma vitamin D levels was not found in patients with and without atrial fibrillation after coronary artery bypass surgery, research hypothesis is rejected. However, due to the limited data collected during this time, it is recommended to conduct other studies with larger sample size for the reliability of the results and to compare them with the results of this study.
